# Supplementary material for: Tree reconciliation combined with subsampling improves large scale inference of orthologous group hierarchies
Source: BMC Bioinformatics. 2019 May 6;20:228. doi: 10.1186/s12859-019-2828-z (PMC6501302; doi:10.1186/s12859-019-2828-z)
Supplement: Supplementary file 1 — Fully expanded OG networks for proteins annotated with InterPro entry IPR013241. Circles represent individual OG, scaled for size (no. of proteins, also shown as label), connected to represent protein overlap to OG at different taxonomic levels (legend). Version 4.0 shows the original OG network with hierarchical inconsistencies while the other methods (RANDOM, v4.5, LS) show the repaired and hierarchically consistent OGs. (PDF 665 kb) [file 12859_2019_2828_MOESM1_ESM.pdf]

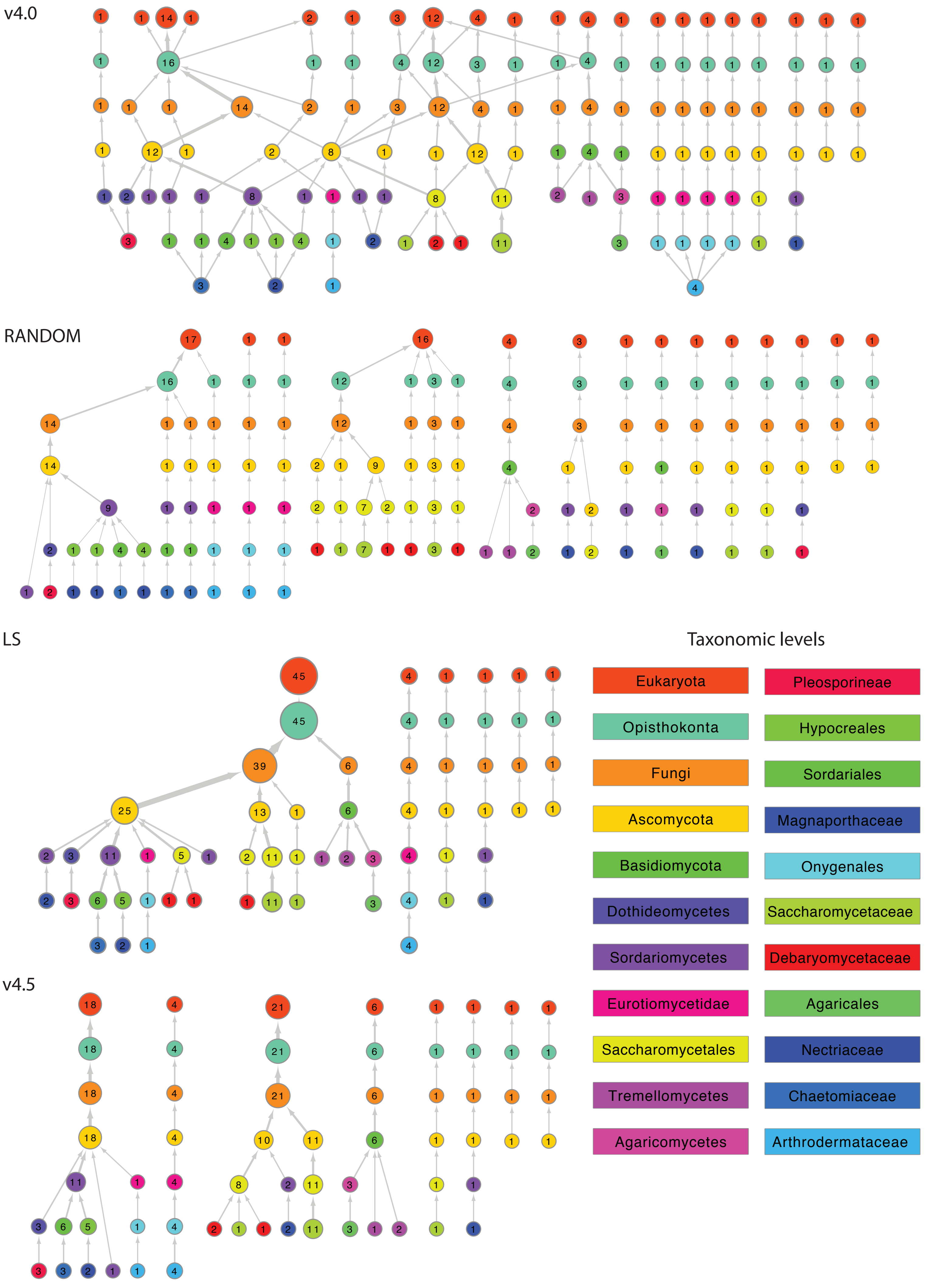
v4.0
RANDOM
LS
v4.5
Taxonomic levels
Eukaryota
Pleosporineae
Opisthokonta
Hypocreales
Fungi
Sordariales
Ascomycota
Magnaporthaceae
Basidiomycota
Onygenales
Dothideomycetes
Saccharomycetaceae
Sordariomycetes
Debaryomycetaceae
Eurotiomycetidae
Agaricales
Saccharomycetales
Nectriaceae
Tremellomycetes
Chaetomiaceae
Agaricomycetes
Arthrodermataceae
