# Supplementary material for: Tree reconciliation combined with subsampling improves large scale inference of orthologous group hierarchies
Source: BMC Bioinformatics. 2019 May 6;20:228. doi: 10.1186/s12859-019-2828-z (PMC6501302; doi:10.1186/s12859-019-2828-z)
Supplement: Supplementary file 2 — Domain benchmark results for sub-types of InterPro annotation and overall OG sizes. (A’-A”) Cumulative results for separated domain sub-types, InterPro Domain type (n=1977) and InterPro Family type (n=2143). For every OG definition (columns), for each domain, the best matching OG (F1 score) across all tested taxonomic levels is chosen. One-sided paired Wilcoxon signed rank test, alternative hypothesis F1(v4.0) - F1(LS) < 0, p-value < 0.0001; F1(v4.5) - F1(LS) < 0 for InterPro Family type 0.02 (A”) and non-significant for InterPro Domain type (A’). (B’-B”) Selective comparison on domains that differ more than 0.1 in F1 score between the compared methods (LS, v4.0, v4.5). Every point in the scatterplot represents the F1 score and size of the best matching OG. Subdivided by InterPro domain type: Domains (n=337) and Family (n=505). (C) Overall distribution of OG size (no. of proteins) per version and taxonomic level. (PDF 3865 kb) [file 12859_2019_2828_MOESM2_ESM.pdf]

fig.S2

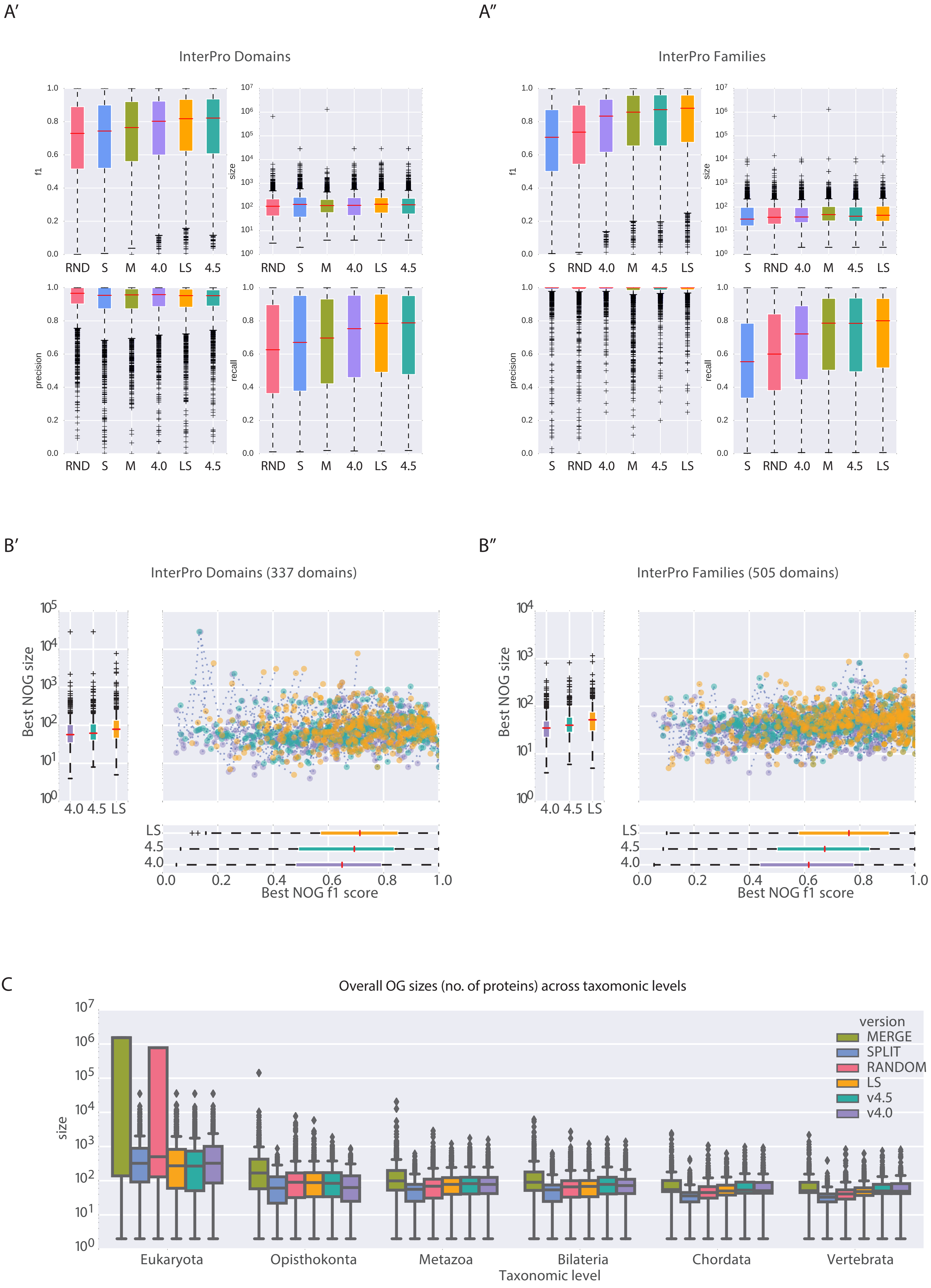
A′
A″
InterPro Domains
InterPro Families
f1
size
precision
recall
RND S M 4.0 LS 4.5
S RND 4.0 M 4.5 LS
B′
B″
InterPro Domains (337 domains)
InterPro Families (505 domains)
Best NOG size
4.0 4.5 LS
Best NOG f1 score
C
Overall OG sizes (no. of proteins) across taxonomic levels
size
version
MERGE
SPLIT
RANDOM
LS
v4.5
v4.0
Eukaryota
Opisthokonta
Metazoa
Bilateria
Chordata
Vertebrata
Taxonomic level
