# Supplementary figures and images for: Tree reconciliation combined with subsampling improves large scale inference of orthologous group hierarchies
Source: BMC Bioinformatics. 2019 May 6;20:228. doi: 10.1186/s12859-019-2828-z (PMC6501302; doi:10.1186/s12859-019-2828-z)

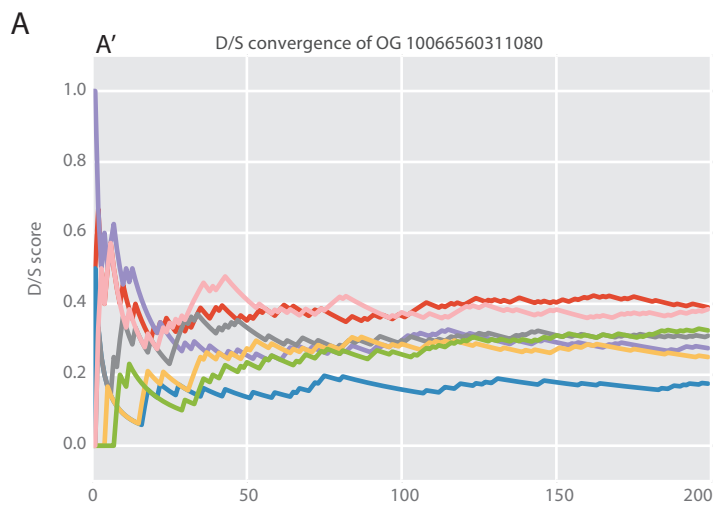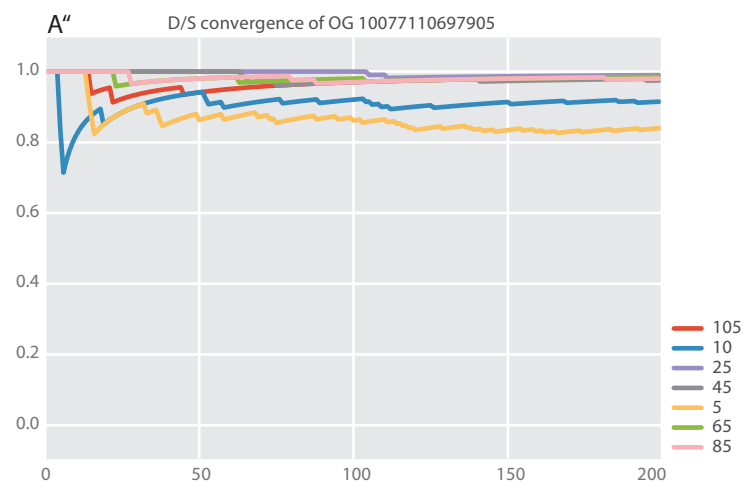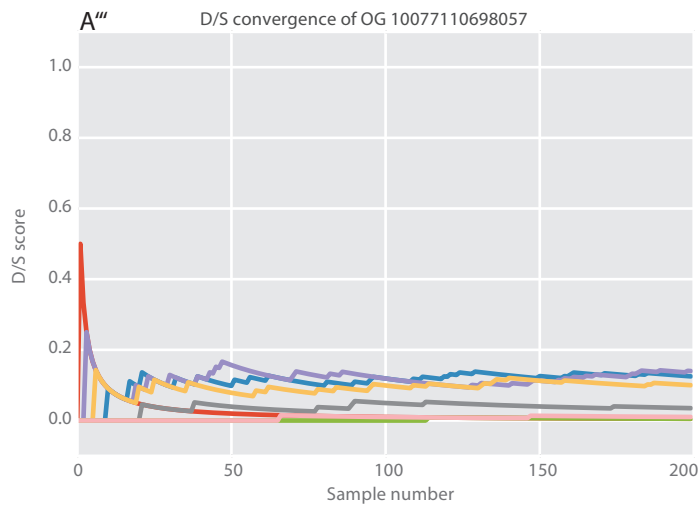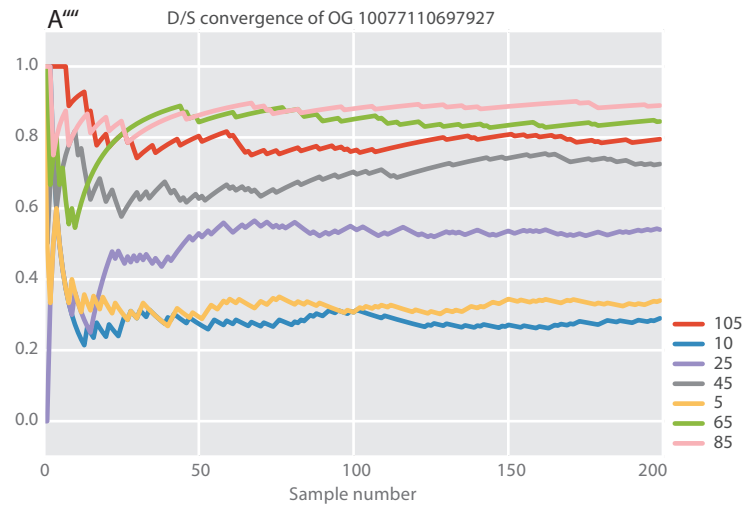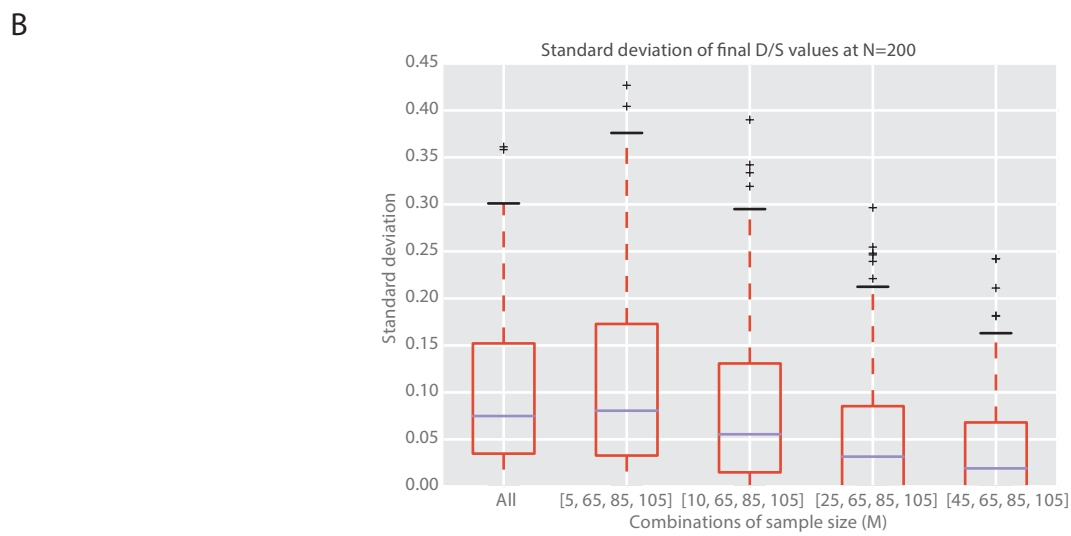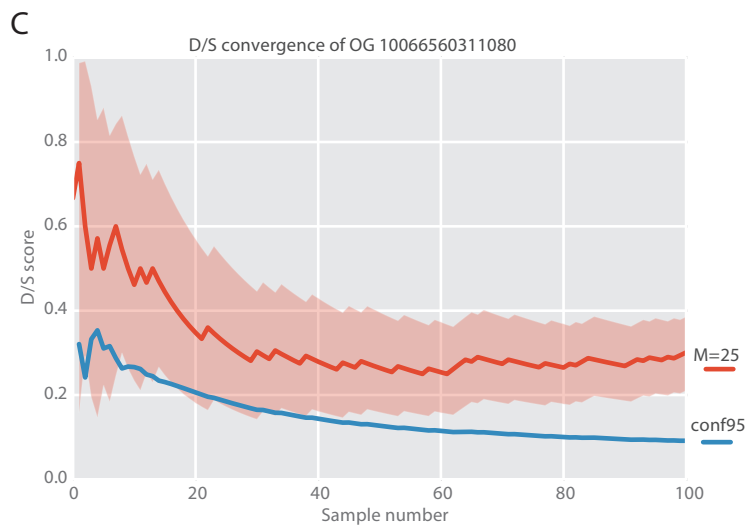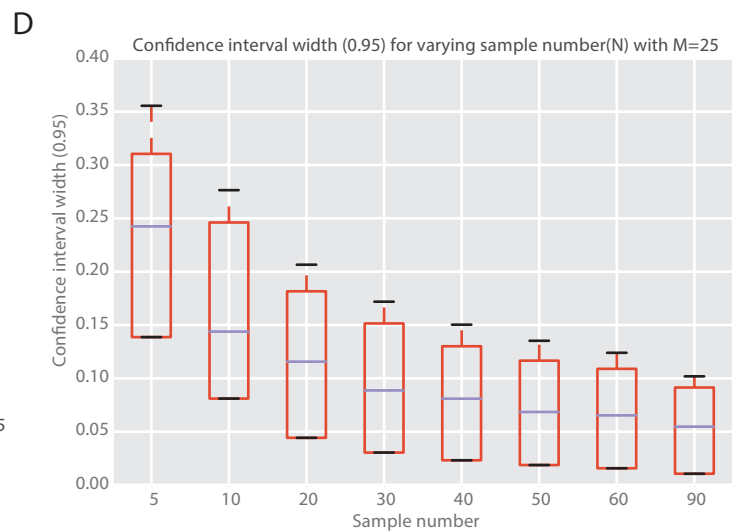

Supplement: Supplementary file 3 — Convergence study for sampling parameters. (A) Duplication-Speciation ratio (D/S) convergence for selected sample sizes (M = 5,10,25,45,65,85,105) over 200 samplings (N). A’-A”’ show examples of solution convergences with low variation across M; A”” shows an example with large variation. (B) Quantification of D/S variation across all tested OGs (n=208) at N=200 for different M combinations. The first including all M values and the other three large values (65,85,105) combined with one smaller value (5,10,25,45). (C) Confidence quantification for D/S of M=25 across increasing N values for example A’ using an exact estimation for binomial processes [33]. (D) Quantification of the 95 percent confidence interval width over all tested OGs (n=208) for M=25 across increasing N values. (PDF 516 kb) [file 12859_2019_2828_MOESM3_ESM.pdf]

Fig.S4

A

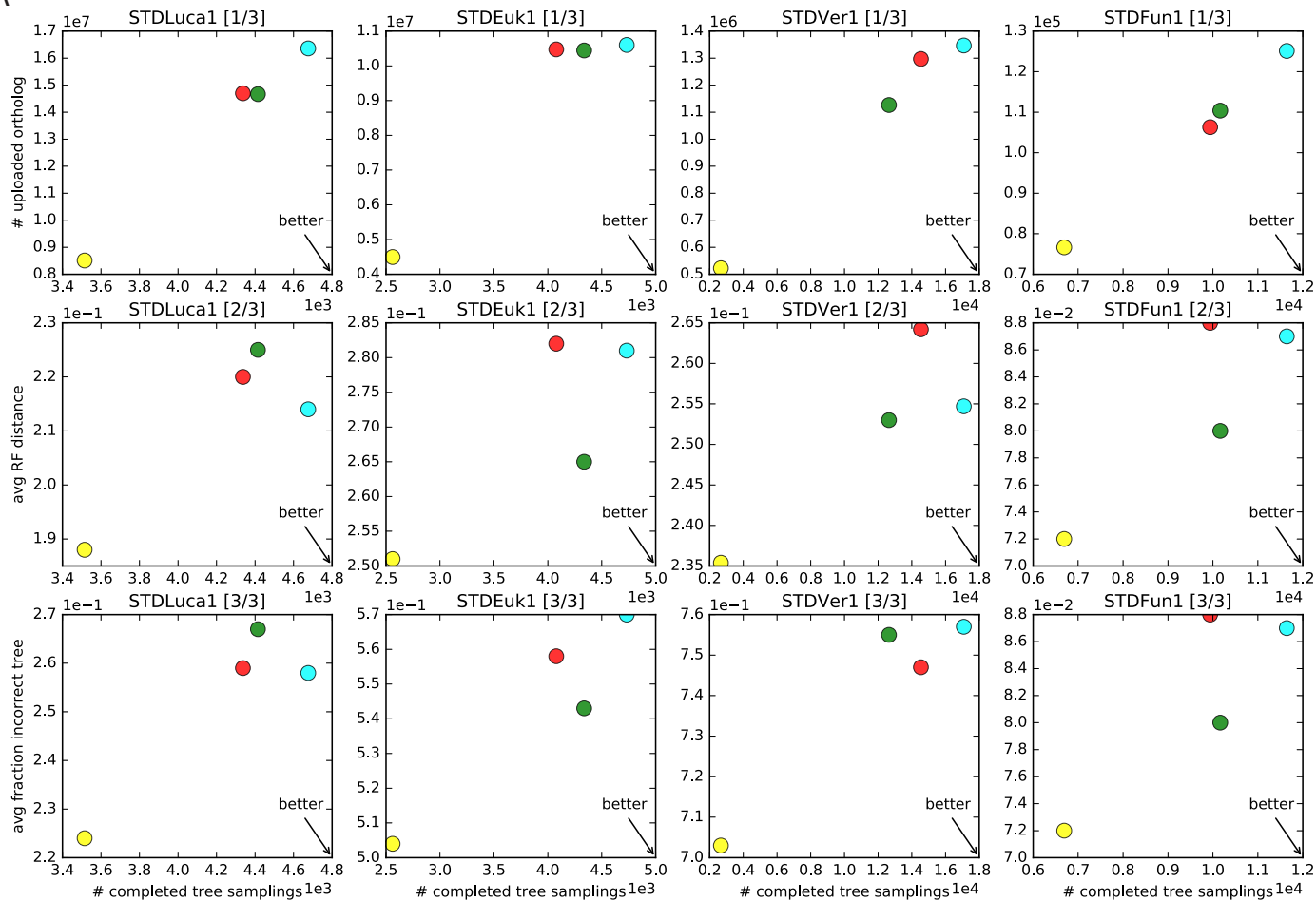

B

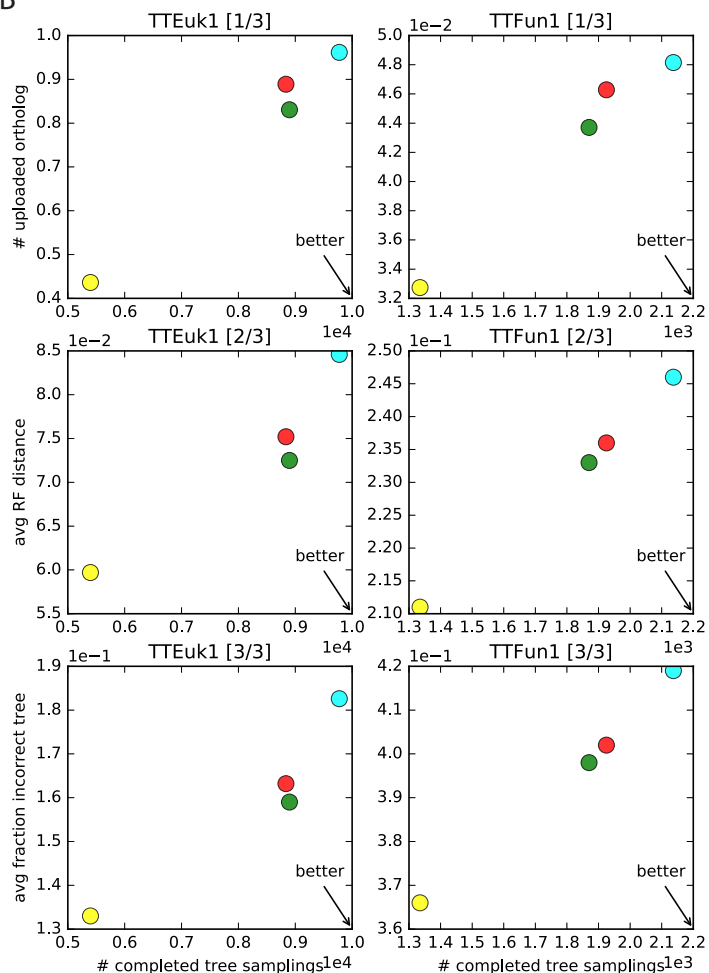

C

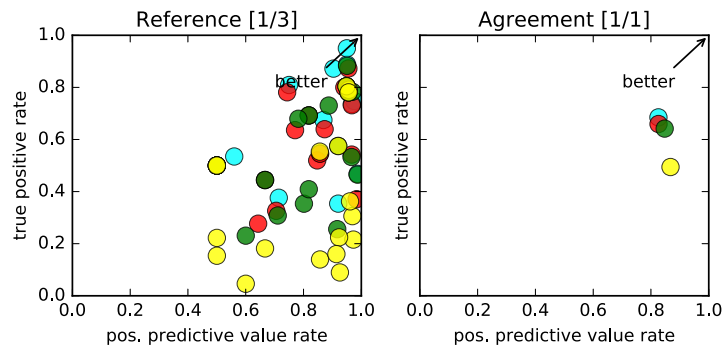

D

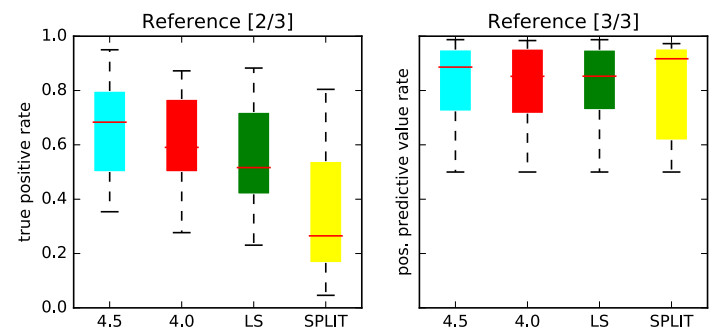

E

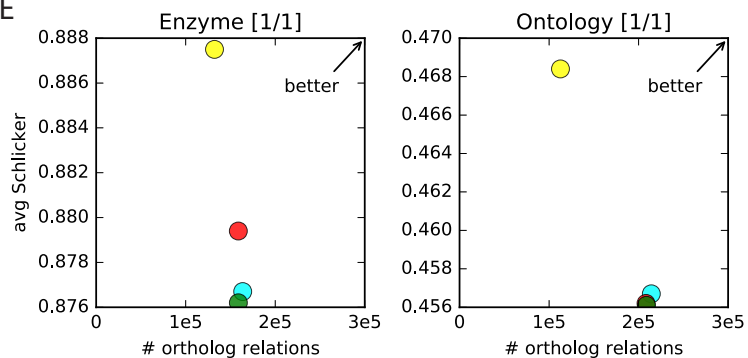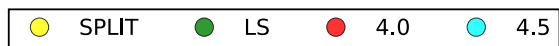

Supplement: Supplementary file 4 — Quest for Orthologs Benchmark. (A) Generalized Species Tree Discordance Benchmark (Altenhoff2016), STD-LUCA [last common universal ancestor]/ Euk[Eukaryotes]/ Ver[Vertebrata]/ Fun[Fungi]; (B) Species Tree Discordance Benchmark (Altenhoff2009); columns distinguish realm (TT-Euk [Eukaryotes]/Fun[Fungi]); (C) Agreement with Reference Gene Phylogenies: SwissTree (Boeckmann 2011); (D) Agreement with Reference Gene Phylogenies: TreeFamA (Li 2006); (E) boxplot for true positive rate in (F); (F) boxplot for positive predictive value rate in (C); (G) Gene Ontology conservation test; (H) Enzyme Classification (EC) conservation test. (PDF 726 kb) [file 12859_2019_2828_MOESM4_ESM.pdf]
