# Supplementary material for: Tree reconciliation combined with subsampling improves large scale inference of orthologous group hierarchies
Source: BMC Bioinformatics. 2019 May 6;20:228. doi: 10.1186/s12859-019-2828-z (PMC6501302; doi:10.1186/s12859-019-2828-z)
Supplement: Supplementary file 5 — Supplementary information for the Quest for Orthologs benchmark reference proteomes mapping process. (PDF 59 kb) [file 12859_2019_2828_MOESM5_ESM.pdf]

Supplementary Information

Mapping of proteomes between QFO benchmark and eggNOG

Using the NCBI taxonomy table (ftp://ftp.ncbi.nih.gov/pub/taxonomy/taxdump.tar.gz) we matched the species by their taxonomy identifier, official name or by best suitable alternative (see TABLE). Subsequently we ran DIAMOND *blastp* [1] to match individual proteins by reciprocal best hit (matching ratio median 0.98; max 1.0; min 0.78; 1.5IQR 0.88).

Conversion algorithm for orthologous groups to pairwise orthologs

To obtain pairwise orthologs for a pair of species in the QFO benchmark we (1) select the best fitting taxonomy level offered by eggNOG (e.g. for human and mouse it is superprimates/spriNOG), (2) filter the OGs of the best fitting level to the once containing both species, (3) generate all possible pairs between the genes of the two species in each OG. This simple procedure relies on the assumption that eggNOG has sufficient resolution in terms of taxonomic level for each species pair, such that the proteins in the relative OGs are indeed orthologs. Because this is not always possible we are aware of the method's limitations and consider the optimization of this problem beyond the scope of this paper.

Table of manually selected mappings between QFO species and eggNOG species

qfo_id	qfo_name	eggNOG_id	eggNOG_name	ncbi_id
5207	cryptococcus neoformans	283643	cryptococcus neoformans var. neoformans b-3501a	5207
83333	escherichia coli	511145	escherichia coli str. k-12 substr. mg1655	83333
173	leptospira interrogans	267671	leptospira interrogans serovar copenhageni str. fiocruz l1-130	173
6945	ixodes scapularis	6945	ixodes scapularis	6945
76856	fusobacterium nucleatum	190304	fusobacterium nucleatum subsp. nucleatum atcc 25586	76856
478009	halobacterium salinarum	64091	halobacterium sp. nrc-1	478009
184922	giardia intestinalis	184922	giardia lamblia atcc 50803	184922
5476	candida albicans	573826	candida dubliniensis cd36	5476
1773	mycobacterium tuberculosis	83332	mycobacterium tuberculosis h37rv	1773
375	bradyrhizobium japonicum	1037409	bradyrhizobium japonicum usda 6	375

Supplementary references

[1] Buchfink B., Xie C., D. Huson, "Fast and sensitive protein alignment using DIAMOND", Nature Methods 12, 59-60 (2015)
